# The histologic features, molecular features, detection and management of serrated polyps: a review

**DOI:** 10.3389/fonc.2024.1356250

**Published:** 2024-03-07

**Authors:** Jin-Dong Wang, Guo-Shuai Xu, Xin-Long Hu, Wen-Qiang Li, Nan Yao, Fu-Zhou Han, Yin Zhang, Jun Qu

**Affiliations:** ^1^ Department of General Surgery, Peking University Aerospace School of Clinical Medicine, Beijing, China; ^2^ Department of General Surgery, Aerospace Center Hospital, Beijing, China

**Keywords:** hyperplastic polyps, sessile serrated lesions, traditional serrated adenoma, histologic features, molecular features, detection, management

## Abstract

The serrated pathway to colorectal cancers (CRCs) is a significant pathway encompassing five distinct types of lesions, namely hyperplastic polyps (HPs), sessile serrated lesions (SSLs), sessile serrated lesions with dysplasia (SSL-Ds), traditional serrated adenomas (TSAs), and serrated adenoma unclassified. In contrast to the conventional adenoma–carcinoma pathway, the serrated pathway primarily involves two mechanisms: BRAF/KRAS mutations and CpG island methylator phenotype (CIMP). HPs are the most prevalent non-malignant lesions, while SSLs play a crucial role as precursors to CRCs, On the other hand, traditional serrated adenomas (TSAs) are the least frequently encountered subtype, also serving as precursors to CRCs. It is crucial to differentiate these lesions based on their unique morphological characteristics observed in histology and colonoscopy, as the identification and management of these serrated lesions significantly impact colorectal cancer screening programs. The management of these lesions necessitates the crucial steps of removing premalignant lesions and implementing regular surveillance. This article provides a comprehensive summary of the epidemiology, histologic features, molecular features, and detection methods for various serrated polyps, along with recommendations for their management and surveillance.

## 1 Introduction

Colorectal cancer (CRC) ranks as the second most prevalent cause of cancer-related mortality and the third most frequently occurring cancer globally. The predominant progression of CRCs occurs through either the conventional adenoma–carcinoma pathway or the serrated pathway, with a smaller proportion originating in the mucosal domain of the gut-associated lymphoid tissue via the third pathway (1).

In contrast to the conventional adenoma–carcinoma pathway, the serrated pathway to CRCs exhibits a potentially accelerated progression and a higher prevalence of CRCs, ranging from 15% to 30%. This highlights the critical significance of detecting and effectively managing serrated lesions (2). Serrated lesions exhibit a distinctive serrated structure in both the epithelium and gland, and can be categorized into several subtypes including Hyperplastic Polyps (HP), Sessile Serrated Lesions (SSL), SSL with dysplasia (SSL-D), Traditional serrated adenomas (TSA), and a newly identified subtype known as serrated adenoma unclassified.

## 2 Serrated polyps

The prevalence of serrated polyps (SP) does not exhibit an age-related increase and is not influenced by sex. However, there may be a correlation between SPs located in the right colon and a family history of CRCs or polyps. Additionally, individuals of White race are at a higher risk of developing SPs, specifically SSLs and Microvesicular Hyperplastic Polyps (MVHP), whereas African Americans and Hispanics have a lower risk of SPs (3, 4).

The development of SPs is influenced by lifestyle factors, particularly smoking, which is strongly associated with the occurrence of SPs, especially the HPs located on the left side of the colon. Moreover, the consumption of alcohol, red meat, fatty acids, and calcium has been positively correlated with an elevated risk of SPs, whereas the intake of NSAIDs/aspirin, cereal fiber, and vitamin D has been inversely associated with the risk. Additionally, individuals with a body mass index ≥ 30 exhibit a 27% higher risk of SPs in the left colon compared to those with a normal weight (4–6).

However, in a study examining the prevalence of HPs, it was found that there was a three-fold increase in prevalence from the “<30 years” age group to the “>69 years” age group (3). In contrast, the prevalence rates of SSLs exhibit minimal variation across different age groups, while the association between prevalence and sex remains a topic of ongoing debate (7, 8). Recent empirical findings indicate that approximately 55% of SSLs are observed in women, and among the 137 instances of SSL-Ds or SSLs with carcinoma, 61% were reported in women (9). When considering TSAs, they typically occur in older patients (typically over 50 years of age) and do not show a significant preference for either gender (10).

**Figure 1:**
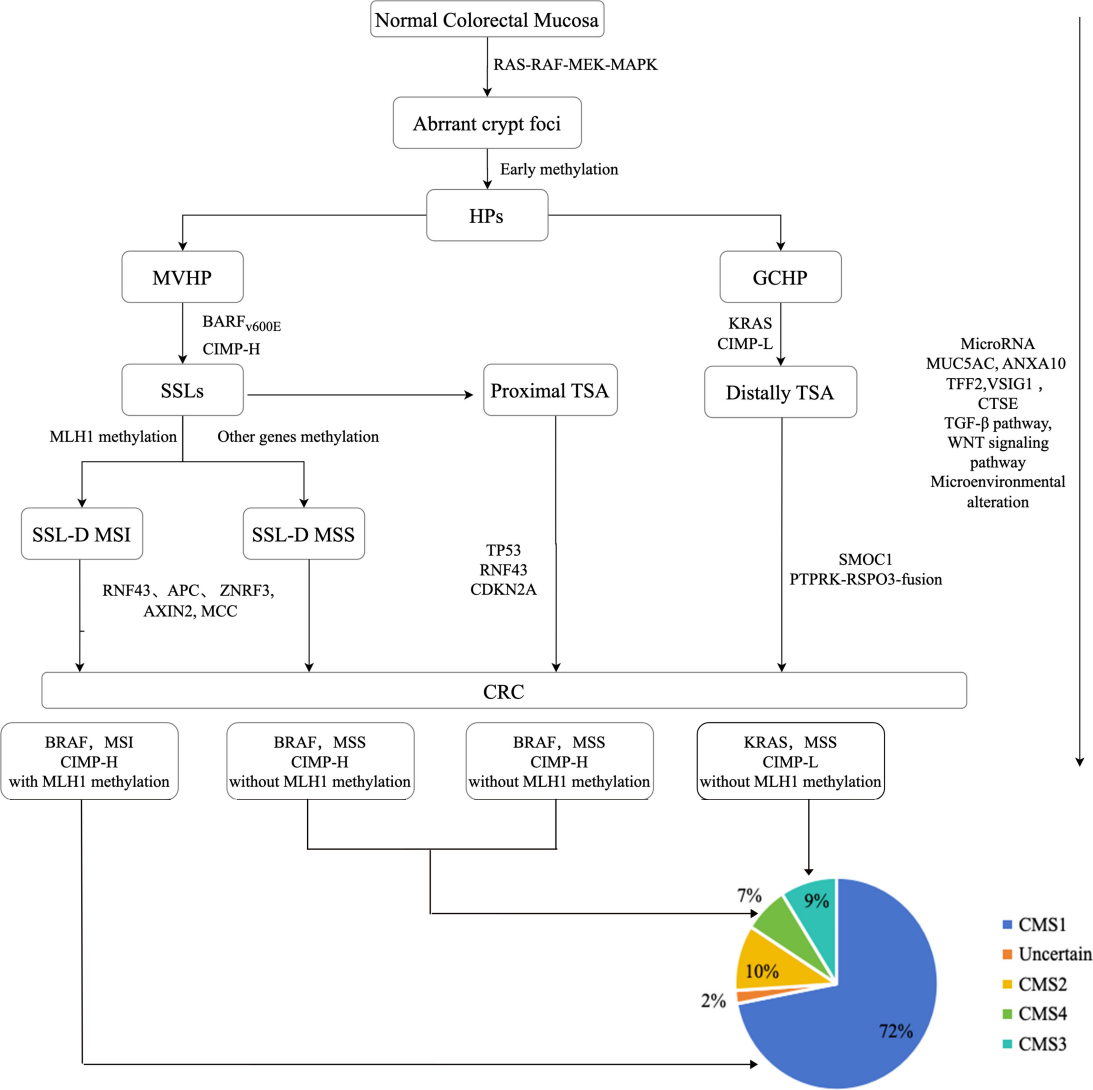
The molecular mechanism of serrated pathway.

In contrast to conventional adenomas, the initial occurrence in serrated precursor lesions involves BRAF/KRAS mutations and hypermethylation, as depicted in **Figure 1**. This leads to the continuous activation of the MAPK signaling cascade, resulting in the disruption of crypt cell proliferation and differentiation. The CpG island methylator phenotype (CIMP) is recognized as the primary mechanism in the serrated pathway, causing the inactivation of several tumor suppressor genes and propelling the development of CRCs (9, 11, 12).

SPs rarely exhibit bleeding, even in advanced stages, thus resulting in a significantly low sensitivity of the fecal immunochemical test (FIT) for detecting SPs. However, the sensitivity can be enhanced by employing multitarget stool DNA tests that incorporate the conventional fecal hemoglobin detection used in FIT along with specific mutation and hypermethylation markers for CRCs. Nevertheless, the widespread adoption of these tests may be hindered by the associated high expenses and logistical complexities (13).

Colonoscopy is considered the gold standard for the detection and resection of polyps, although up to 20% of polyps are missed during colonoscopy (13). It is crucial to acknowledge that diverse endoscopic techniques possess distinct characteristics and may demonstrate variations in their diagnostic efficacy (**Table 1**) (14). In contrast to white light endoscopy, chromoendoscopy entails the application of a contrast dye solution, which is either sprayed or irrigated onto the colonic mucosa.This technique serves to enhance the identification, examination, characterization, and thorough removal of mucosal lesions. A singular study demonstrated a notable enhancement in the detection rate of serrated lesions when employing chromoendoscopy as opposed to conventional colonoscopy (15). Nonetheless, the existing body of research in this particular area remains limited, thus warranting further investigation. Narrow band imaging (NBI) is a technique that uses narrow wavelength light source to optimize the visualization of hemoglobin, seen as microvessels in the mucosa. Numerous studies compare white light and NBI in detecting polyps. Based on prior research, the detection rate of SSL is estimated to be around 7.5% with NBI and varies between 6.8% and 8.0% with white light endoscopy, although no statistically significant difference has been observed (15–18). NBI is believed to be more effective for visual diagnosis rather than polyp detection.

**Table 1:** The different endoscopic modalities.

Diagnostic method	Characteristic
White-Light Endoscopy	➢ The gold standard for the detection
Chromoendoscopy	➢ Spraying dye ➢ More visible and higher detection rate ➢ Results in more biopsies ➢ Cost increase ➢ No side effects
NBI	➢ Show the microvasculature of the mucosa. ➢ NICE classification differentiate SPs from adenomas ➢ WASP features help distinguish SSLs from HPs. ➢ Determine whether SSLs may have foci of dysplasia

### 2.1 Hyperplastic polyps

HPs, previously referred to as metaplastic polyps, constitute 30% of all colorectal polyps and approximately 75% of all SPs. It is commonly believed that HPs are non-malignant lesions. Nevertheless, certain HPs (especially those larger than 5 mm and/or located in proximal regions) have the potential to develop into SSLs and SSL-Ds, ultimately leading to CRCs (9, 19).

The two types of HPs, namely the mucin-vacuolated HP (MVHP) and the goblet cell-rich HP (GCHP), are distinguished based on their pathological characteristics. MVHPs are more prevalent compared to GCHPs. Previously, the mucin-depleted type was regarded as a separate subset; however, it is now understood to arise from regenerative alterations in other HPs (20, 21).

#### 2.1.1 Histologic features

**Figure 2:**
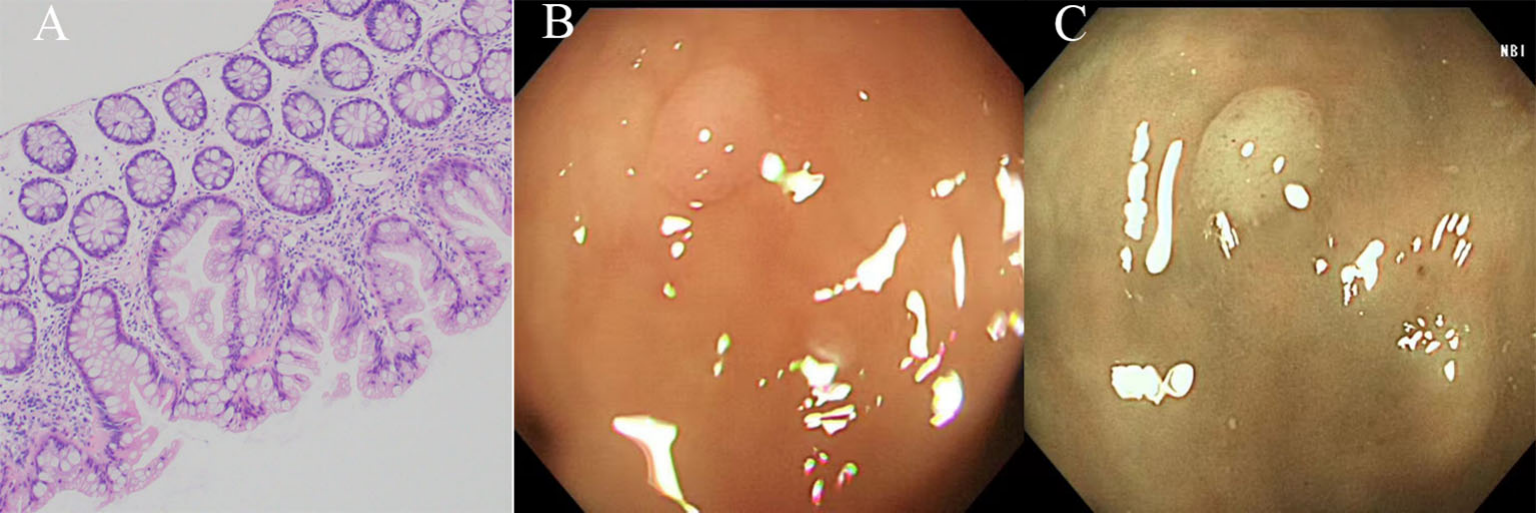
The histological and endoscopic images of hyperplastic polyps. **(A)** The histological images of hyperplastic polyps. **(B)** The white light endoscopic images of hyperplastic polyps. **(C)** The NBI images of the hyperplastic polyps.

The histological characteristics of HPs predominantly involve uniform hyperplasia of the epithelium in the upper two-thirds of the saphenous fossa. This results in the formation of small papillae that extend into the lumen of the saphenous fossa, thereby giving the luminal surface a serrated appearance (**Figure 2A**). The diagnosis of HPs requires the presence of a distinctive serrated shape in the epithelium, while lacking the histological criteria associated with SSLs (13, 22).

MVHPs exhibit small droplets of mucin, stellate lumina within the crypts, and microvesicular epithelial cells with ample cytoplasm. Conversely, GCHPs display an elevated count of goblet cells and possess a more inconspicuous serrated appearance resembling surface tufting, thereby posing challenges in distinguishing GCHPs from normal mucosa. It is worth noting that the differentiation between MVHPs and GCHPs holds no clinical significance (9, 20, 23).

#### 2.1.2 Molecular features

According to the hypothesis presented in **Figure 1**, MVHPs and SSLs are believed to exhibit the same progression pattern due to the presence of BRAF V600E mutation and CIMP-High (CIMP-H). Conversely, GCHPs are posited as precursors to TSA, characterized by KRAS mutation and CIMP-Low (CIMP-L). In terms of genomic methylation, MVHPs and left-sided SSLs demonstrate low to intermediate levels, whereas right-sided SSLs exhibit high levels of methylation. Both the MVHP-SSL and GCHP-TSA pathways are potential routes to CRCs within the serrated pathway, and there is a possibility of overlap between them. However, the question of whether MVHPs and SSLs arise independently has not yet been definitively answered (12, 24).

The MUC family, consisting of MUC1 to MUC24, encompasses glycoproteins that play crucial roles in cellular signaling and barrier defense. Within MVHPs, a notable hypomethylation of the MUC5AC gene is observed, while benign HPs exhibit a deficiency in MUC6 expression (12, 25).

#### 2.1.3 Detection

HPs are typically small in size (≤5 mm) and are primarily found in the distal colon, particularly in the rectum. In addition, MVHPs are predominantly observed in the right side of the colon and they have a broader distribution compared to GCHPs (12, 26).

In white light endoscopy, HPs manifest as roundish pale flat-elevated or sessile formations, occasionally exhibiting normal mucosal coverage (**Figure 2B**). Chromoendoscopy reveals the presence of Kudo type II asteroid pits on the surface of these lesions. NBI demonstrates that HPs exhibit the same color as the surrounding mucosa and lack prominent vessels on their surface. (**Figure 2C**, Nice type1) (12, 13).

### 2.2 Sessile serrated lesions

SSLs, previously referred to as sessile serrated adenoma/sessile serrated polyp, were reclassified by the World Health Organization in 2019 due to the presence of some SSLs lacking dysplasia on histology and polypoid morphology. SSLs play a significant role as precursors to CRCs, constituting 3.9% of all colorectal polyps and 15-25% of all SPs (3, 13).

SSL-Ds serve as an intermediary stage within the serrated pathway, bridging the gap between SSLs and invasive CRCs. These lesions are relatively uncommon, with an estimated occurrence rate of approximately 0.5% among average-risk patients, accounting for approximately 4-8% of all SSLs (9). It is hypothesized that these infrequent lesions exhibit a brief period of residence during the transition from dysplasia to carcinoma (27).

#### 2.2.1 Histologic features

**Figure 3:**
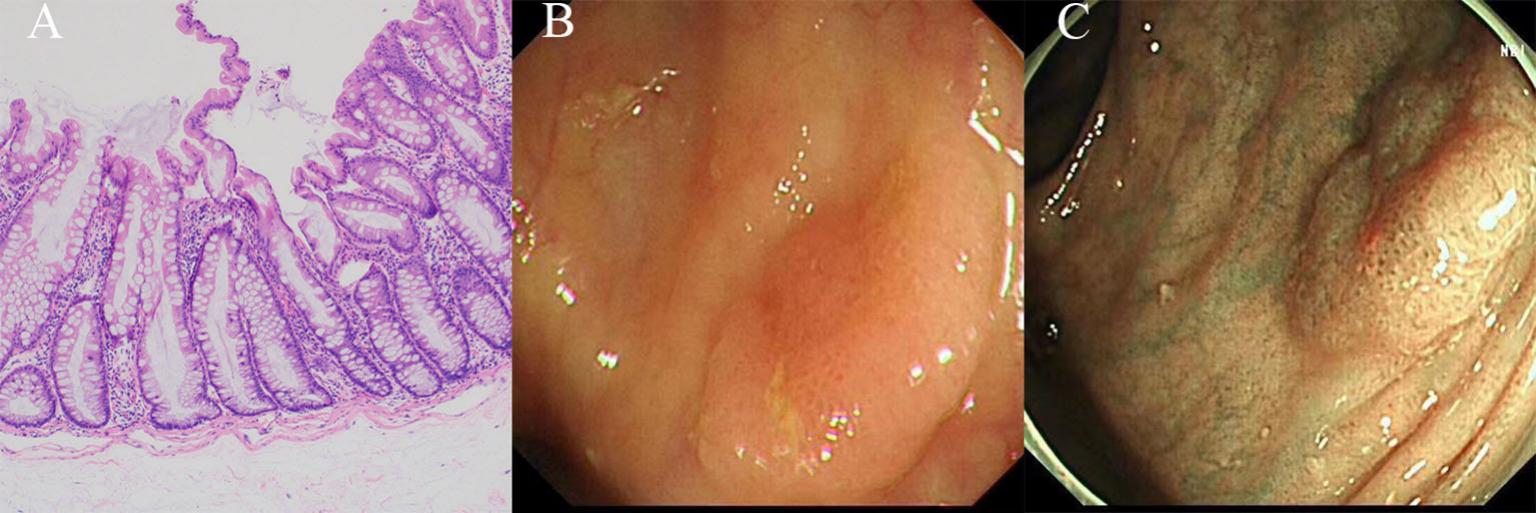
The histological and endoscopic images of sessile serrated lesions. **(A)** The histological images of sessile serrated lesions. **(B)** The white light endoscopic images of sessile serrated lesions. **(C)** The NBI images of the sessile serrated lesions.

SSLs exhibit a combination of goblet and non-goblet cells observed in the crypt bases, with a slightly more prominent presence compared to HPs. Moreover, SSLs manifest as a flat elevated hyperplastic mucosa, characterized by a distinctive morphological alteration in the form of a “boot” or “inverted T” shape at the crypt bottom (**Figure 3A**). The diagnosis of SSLs necessitates the identification of at least one abnormal crypt. A fundamental distinction from HPs is that the entire saphenous fossa demonstrates serration and grows horizontally along the mucosal muscular layer, specifically within the third of the crypt basal expansion. Dysplasia is not observed in uncomplicated SSLs, however, SSLs with areas of conventional (tubular or tubulovillous) adenoma-like dysplasia may indicate progression towards carcinoma (9, 20, 22, 28).

In comparison to SSLs, SSL-Ds exhibit greater histological heterogeneity. SSL-Ds often exhibit a polypoid morphology, while the non-dysplastic portion of the same lesion appears flat, making it challenging to discern the extent of hyperplasia. The surrounding glands adjacent to the SSL-Ds exhibit villous structures that are longer and more densely packed, displaying complex branching, sieve-shaped crypts, and increased or decreased serration compared to those in SSLs. Furthermore, the utilization of immunohistochemical analysis for MLH1 is crucial in the determination of the presence of clinically significant dysplasia within SSLs (9, 20, 22).

The dysplasia observed in SSL-Ds can be categorized into various subtypes, including adenomatous/intestinal dysplasia, serrated dysplasia, minimal deviation dysplasia, and dysplasia not otherwise specified. Adenomatous dysplasia, although relatively uncommon, shares similarities with dysplasia found in conventional adenomas. It is characterized by the sustained expression of MLH1 and appears to exhibit limited progression to colorectal cancer, particularly in cases of low-grade dysplasia. Serrated dysplasia is a more common occurrence characterized by the presence of eosinophilic cytoplasm and small crowded glands exhibiting notable nuclear atypia and mitotic activity. The loss of MLH1 staining is not commonly observed, but its presence can be interpreted as an indication of progression to TSA. In addition, minimal deviation dysplasia showcases only slight cytological and architectural alterations, yet it is predominantly accompanied by a loss of MLH1 expression (91%) (12, 13, 20, 29).

It is not advisable to assess the severity of dysplasia occurring in SSL-Ds, as even SSLs with low-grade dysplasia may have a considerably elevated likelihood of progressing to CRC (30).

#### 2.2.2 Molecular features

The BRAF V600E mutation, in conjunction with CIMP-H, is recognized as a molecular characteristic within the colorectal sessile serrated neoplasia pathway. The prevalence of the BRAF mutation in SSLs ranges from 70% to 81%, whereas the occurrence of KRAS mutation (approximately 9% of SSLs) is considerably less frequent (9).

CIMP of tumor suppressors, such as MLH1 and p16INK4a, leads to the silencing of these genes and facilitates the progression of SSLs to SSL-Ds (**Figure 1**). SSL-Ds with MLH1 hypermethylation are known to evolve into microsatellite instable-high (MSI-H) colorectal cancers (CRCs) with immune activation, while SSL-Ds without MLH1 alteration are associated with the development of microsatellite stable (MSS) CRCs with immune suppression. Approximately 75% of SSL-Ds exhibit a loss of MLH1 staining in dysplastic regions, indicating MLH1 hypermethylation. Furthermore, when comparing the MSS SSL-Ds and CRCs to the MSI SSL-Ds and CRCs, it is evident that the latter exhibit notably elevated levels of intra-epithelial lymphocytes (IELs) density values and PD-1/PD-L1 expression, indicating a significant difference. Consequently, MLH1 methylation and IELs counts can be considered as autonomous and dependable factors in the identification of SSL-Ds. Notably, the emergence of MSS CRCs from SSLs does not necessarily necessitate mutations in MAPK pathway genes for their formation (9).

The WNT signaling pathway is known to have a significant impact on the dysplastic changes observed in SSLs. Truncating mutations in RNF43, APC, and ZNRF3 are prevalent in over 60% of SSL-Ds, while mutations in genes associated with the WNT signaling pathway are rare in SSLs. This observation is supported by the presence of nuclear β-catenin accumulation and MYC overexpression in most SSL-Ds, but not in SSLs. Furthermore, SSLDs with MSI-H have been found to exhibit a high mutational rate of FBXW7 and alterations in WNT-pathway-associated genes such as RNF43, APC, AXIN2, and MCC, as reported in previous studies (9, 12, 19).

The progression of SSLs to CRCs is influenced by the combined effects of CIMP of tumor suppressor, activation of WNT signaling, and microenvironmental changes. Additionally, the development of CRCs can be facilitated by microbiota and miRNAs. A study has observed an increased abundance of the genus Fusobacterium in individuals with serrated lesions. Furthermore, there is a significant difference in miRNA expression between the serrated and conventional pathways in colorectal carcinogenesis. Specifically, miRNA-31 expression has been found to be associated with CIMP status in serrated lesions with BRAF mutation. However, the precise role of miRNA in this process remains unclear (11, 31–33).

The expression of Agrin (AGRN) in the muscularis mucosa appears to be a distinctive characteristic of SSLs. Furthermore, the presence of LOH or promoter hypermethylation of SLIT2 has been identified as an additional molecular marker for SSLs. Other potential molecular biomarkers observed to be overexpressed in SSLs include cathepsin E (CTSE), trefoil factor 1, trefoil factor 2 (TFF2), v-set and immunoglobulin domain containing 1 (VSIG1), annexin A10 (ANXA10), and MUC5AC. Conversely, Hes-1 is downregulated in SSLs when compared to normal tissues and HPs (12).

#### 2.2.3 Detection

As previously mentioned, colonoscopy is widely regarded as the preferred method for detecting polyps. However, the present identification of SSLs during colonoscopy is frequently inadequate and relies heavily on histopathological diagnosis following biopsy or resection (13).

SSLs and SSL-Ds are commonly found in the proximal colon, specifically on the right side, and are typically larger than 5 mm in size. SSLs often exhibit faint borders and a pale surface, along with a mucus cap, making it challenging to differentiate them from the surrounding mucosa (**Figure 3B**). This difficulty in distinguishing SSLs can lead to missed or delayed diagnoses and incomplete resections. In chromoendoscopy, SSLs resemble HPs but display a Kudo type II-O pattern and dark spots caused by dilated crypts. NBI (Nice type 1) can reveal varicose microvascular vessels running throughout the deep mucosal layer (**Figure 3C**). The diagnostic characteristics of SSLs include inconspicuous borders, cloud-like surfaces, dark spots, and varicose microvascular vessels (9, 12, 13, 22).

In contrast, SSL-Ds are frequently characterized by a pedicled, bimodal appearance, central depression, and reddish color. When observed under white light endoscopy, most SSL-Ds are likely to display either large or small nodules on the surface, while chromoendoscopy reveals Kudo type III or Kudo type IV patterns (12, 22).

Differentiating between HPs and SSLs has consistently posed challenges, particularly when the polyps are smaller than 5mm. HPs located on the right side or those exceeding 5mm in size are more likely to be reclassified as SSLs upon re-review. A study demonstrated that a multivariate analysis considering factors such as location (proximal colon), size (≥ 10 mm), glandular opening, and microvascular morphology of the serrated lesions provides a more accurate diagnosis of SSLs compared to a single factor diagnosis (22, 34, 35).

### 2.4 Traditional serrated adenoma

TSAs are a relatively rare type of colorectal polyps, accounting for less than 1% of all cases. Additionally, they represent 1-7% of all serrated polyps and are considered a precursor to CRCs. TSAs exhibit serrated architecture resembling HP and also display dysplastic nuclear changes resembling adenomas. It is worth noting that TSAs can be further classified based on the presence of dysplasia, which can manifest as either “adenomatous dysplasia” or the less frequently observed “serrated dysplasia”. Only the latter subtype has the potential to progress into invasive carcinomas (35–37).

#### 2.4.1 Histologic features

**Figure 4:**
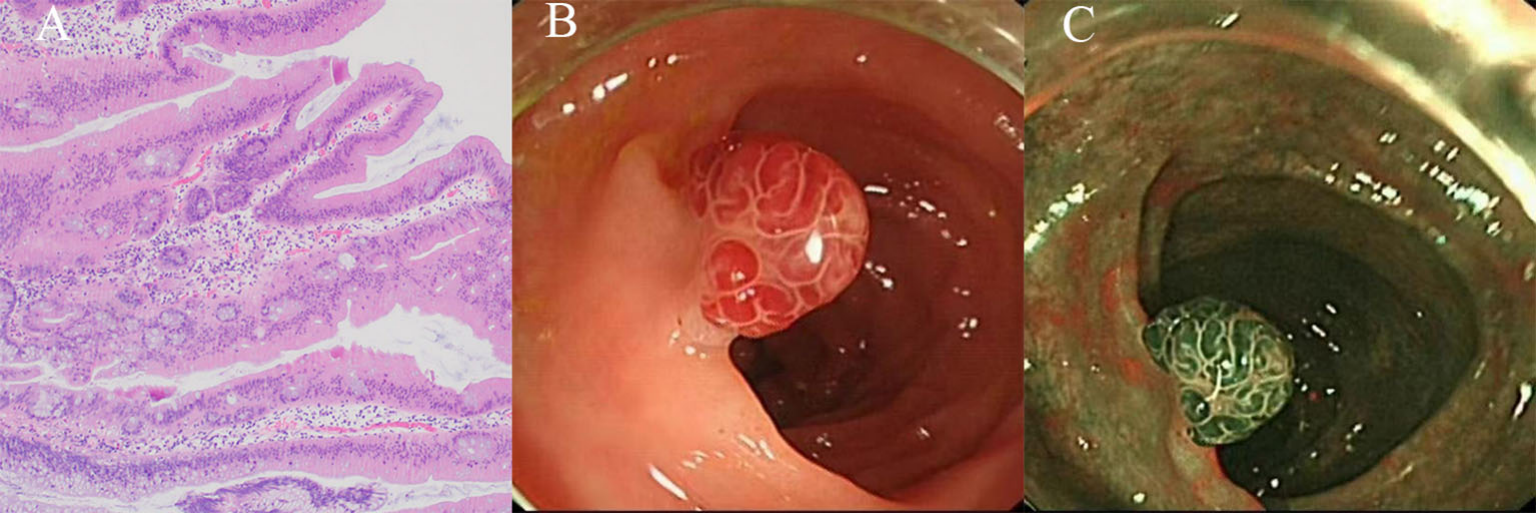
The histological and endoscopic images of traditional serrated adenomas. **(A)** The histological images of traditional serrated adenomas. **(B)** The white light endoscopic images of traditional serrated adenomas. **(C)** The NBI images of the traditional serrated adenomas.

TSAs exhibit villous and occasionally filiform architecture, characterized by cells that display ectopic crypt formation (ECF), slit-like serration, and typical cytology (eosinophilic cytoplasm and elongated pencillate nuclei with delicate dispersed chromatin) (**Figure 4A**). To diagnose a TSA, at least two of these three features must be present, with at least one feature being observed in 50% of the polyp. ECF, which refers to the development of epithelial islets orthogonal to the main crypt axis and unrelated to the muscularis mucosa, is a distinctive characteristic of TSAs and represents the proliferation zones of these polyps. Moreover, slit-like serration is the most consistent histological feature of TSAs (10, 20, 38).

TSAs exhibit a higher count of intraepithelial lymphocytes in comparison to traditional adenomas, albeit significantly lower than that observed in SSL-Ds.10 It is widely acknowledged that TSAs possess inherent dysplastic characteristics, although some TSAs may lack dysplastic features. Regions of dysplasia within TSAs may signify a more severe progression, yet there are presently no established surveillance guidelines for these lesions. Further research on TSAs is imperative to distinguish between typical TSAs and advanced dysplastic TSAs (13, 20).

#### 2.4.2 Molecular features

TSAs can originate from either BRAF-mutant serrated lesions or KRAS-mutant lesions (80%). The presence of gene mutations, coupled with other molecular alterations, facilitates the proliferation of intestinal epithelium and leads to the formation of TSAs with distinctive cytomorphology. Additionally, there exists a smaller subset of TSAs that lack mutations in both BRAF and KRAS genes, and their emergence is currently attributed to unidentified molecular mechanisms (12, 39).

TSAs and GCHPs that are situated distally often exhibit KRAS mutations. TSAs with KRAS mutations commonly display CIMP-L or CIMP characteristics and subsequently develop into MSS CRCs. Furthermore, TSAs originating from the distal colon demonstrate specific methylation of the SMOC1 gene and subsequent loss of its expression, which are frequently associated with high-grade adenoma and CIMP-L/MSS CRCs (12, 19, 39).

Proximal TSAs, particularly those smaller than 10 mm in size, frequently exhibit BRAF mutations and display CIMP-H characteristics, while maintaining MLH1 expression. These TSAs consistently manifest additional mutations in TP53 and other genes associated with cancer, resulting in MSS CRCs. CDKN2A mutation occurs more frequently in TSAs compared to SSLs, particularly in advanced lesions with BRAF mutations. Additionally, SSLs and HPs are commonly found in close proximity to BRAF-mutated TSAs (12, 19, 20, 30).

The WNT signaling pathway is known to have a significant impact on both BRAF-mutant and KRAS-mutant TSAs. However, the activation of the WNT signaling pathway in TSAs differs from that in traditional adenomas, as it is primarily caused by PTPRK-RSPO3 fusions or RNF43 mutations rather than APC inactivation. Additionally, these genetic alterations are almost exclusively observed in TSAs and are mutually exclusive. Specifically, PTPRK-RSPO3 fusions are consistently present in KRAS-mutant TSAs, while RNF43 mutations are more commonly found in BRAF-mutated TSAs. Additional minor fusions, such as NRIP1–RSPO2, EIF3E–RSPO2, and PIEZO1–RSPO2, have also been identified in TSAs. Notably, these mutations are exclusively present in TSAs and not in the adjacent SSLs and HPs, suggesting that the WNT signaling pathway may play a crucial role in the progression from precursor lesions to TSAs (11, 40).

TSAs, similar to SSLs, demonstrate atypical expression of gastric proteins such as MUC5AC, ANXA10, TFF2, VSIG1, and CTSE, albeit with significantly lower levels compared to HPs and SSLs. Currently, there are no established biomarkers specific to TSAs. However, LEFTY1, a protein that suppresses the TGF-β pathway, exhibits overexpression exclusively in TSAs. This finding holds potential for the molecular diagnosis of TSAs (11, 40).

#### 2.4.3 Subtypes of TSAs

Mucin-Rich Traditional Serrated Adenoma (MrTSA) can be defined as a Traditional Serrated Adenoma (TSA) that exhibits a goblet cell or mucin-rich cell composition of at least 50%, with a goblet cell to eosinophilic absorptive cell ratio of 1:1 or higher. MrTSAs are characterized by a reduced occurrence of ECFs, an increased presence of intraepithelial lymphocytes, and the continued presence of undulating serration. Furthermore, MrTSAs exhibit sustained MLH1 expression, frequent BRAF mutations, and infrequent KRAS mutations. These observations suggest that the majority of MrTSAs may serve as precursors to BRAF-mutant MSS CRCs. Additionally, MrTSAs commonly express more MUC5AC in comparison to TSAs (11, 30, 40).

Superficially serrated adenoma (SuSA), which exhibits a combination of adenomatous and serrated features with superficial spread, has been reported to be associated with a specific subtype of TSA. From a molecular perspective, SuSAs frequently exhibit KRAS mutations and RSPO fusions, and they may also serve as precursors for KRAS-mutant MSS CRCs (9, 40, 41).

#### 2.4.4 Detection

TSAs predominantly reside in the distal colon, typically on the left side, and exhibit an average size of approximately 15 mm. They manifest as reddish, protruding, or pedunculated lesions when observed under white light endoscopy, resembling either “pinecone-like” or “branch coral-like” structures in macroscopic examination (**Figure 4B**). In chromoendoscopy, the presence of type IIIH pits, resembling Kudo type IIIL-like tubular pits, or type IVH pits, resembling Kudo type IV-like villous pits, is observed. When using NBI, these pits manifest as superficial or protruding lesions, occasionally exhibiting a pedunculated morphology (**Figure 4C**). Typically, these lesions exceed 5 mm in size and display dilated vessels, characterized by an expanded and brown capillary network resembling leaf vein, predominantly located in the extensive stromal region surrounding the crypts (13, 42, 43).

### 2.5 Unclassified serrated adenoma

The Unclassified serrated adenomas typically exhibit a combination of features found in both serrated and conventional polyps, such as serrated tubulovillous adenomas (sTVA) and superficially serrated adenoma. sTVAs are distinguished by the presence of numerous ECFs, although lacking eosinophilic cytoplasm and undulating serration. Genetically, sTVAs may undergo a phenotypic transition as they accumulate genetic alterations, transitioning from a serrated pathway to a more conventional pathway. The superficially serrated adenoma exhibits a superficial epithelium with serrations, along with proliferative cells that are specifically localized to the middle and lower layers of the mucosa. It is noteworthy that the WNT signaling pathway is active in both the sTVA and superficially serrated adenoma (20, 30, 44, 45).

### 2.6 Serrated polyposis syndrome

Serrated polyposis syndrome (SPS) is a clinical condition characterized by a multitude of SPs. The WHO classification defines two subtypes of SPS (13):WHO2019-I: at least 5 SPs, all ≥5 mm, including at least two SPs ≥10 mm, located proximally to the rectum. WHO2019-II: at least 20 SPs throughout the entire colon, of which at least 5 SPs are located proximally to the rectum.

SPS is the predominant form of polyposis syndrome, encompassing various histological subtypes of serrated polyps such as HPs, SSLs, TSAs, and serrated adenoma unclassified. The prevalence of SPS among individuals undergoing colonoscopy ranges from 0.03% to 0.5%, with no significant gender disparity (12, 46).

SPS is typically diagnosed in individuals in their fifth decade of life, and there are no significant variations in age between Western and Asian countries. The correlation between lifestyle factors and SPS aligns with those observed in SPs. The genetic basis of SPS remains unidentified. Although the majority of SPS cases are not linked to specific genetic variants, a small percentage of patients (<3%) with SPS exhibit a germline mutation in RNF43. Additionally, other genes such as EPHB2, ATM, PIF1, RBL1, TELO2, and XAF1 may potentially contribute to the pathogenesis of SPS (12, 47).

### 2.7 Serrated lesions of the appendix

The appendix’s serrated lesions have been categorized into the subsequent subgroups: hyperplastic polyps, sessile serrated lesions with dysplasia, and sessile serrated lesions without dysplasia. The histological features of appendiceal lesions can exhibit similarities to both HPs and TSAs, while mucinous appendiceal neoplasia demonstrates a high prevalence of KRAS mutations. This suggests a potential association between appendiceal lesions and GCHPs as well as TSAs. Furthermore, the role of the serrated pathway in the progression to carcinoma appears to be less significant in the appendix compared to other regions of the right colon (20, 48–50).

## 3 Management

The primary objective of CRCs screening programs is to detect and eliminate premalignant lesions. Consequently, it is imperative to ascertain the attributes of various serrated lesions and their potential for malignancy in order to effectively manage these lesions and establish appropriate surveillance protocols.

### 3.1 Characteristics of different SPs

The differentiation of various serrated lesions can be challenging, particularly due to the ongoing development of diagnostic criteria. The **Table 2** provides a concise overview of the distinguishing features of different SPs (51).

**Table 2:** The characteristics of different SPs.

	HPs	SSLs	TSAs
Proportion	75%	15-25%	1-7%
Size	Usually ≤ 5 mm	Usually > 5 mm, an average size of 5-7mm	An average size of 15 mm
Location	Usually in the distal Colon(left-sided,70-80%).	Usually in the proximal colon (right-sided,75-90%)	Mostly in the distal colon(left-sided)
Crypt architecture	MVHP: Funnel-shaped,serrations limited to upper two-thirds. GVHP: Elongated,Little to no serrations	Horizontal growth along the muscularis mucosae, dilation (often asymmetric), serrations extending into the crypt base	Slit-like serrations, often ECF
Proliferation zone	Uniformly in the Basal of crypts	May abnormally away from the crypt base	Present within ECF and crypt base
Cytologic features	Small basally located nuclei	Occasional larger nuclei with inconspicuous nucleoli	Pencillate nuclei, Eosinophilic cytoplasm, Dysplasia
Mucin type	MVHP: Microvesicular and Goblet cell GVHP: Goblet cell only	Microvesicular and Goblet cell	Occasional scattered goblet cells; rare goblet cell
Molecular features	MVHP: BRAF mutation (70–80%) GVHP: KRAS mutation (50%)	BRAF mutation (>90%) KRAS mutation (0-5%)	BRAF mutation (20–40%) KRAS mutation (50–70%)
White light Endoscope	Roundish pale flat-elevated Or sessile lesions	Faint borders Pale surface Mucus cap	Reddish, protruded or pedunculated,”pinecone-like” or “branch coral-like”
Chromoendoscopy (Kudo’s classification)	Type II	Type II-O SSL-D: Type III, IV	Type IV-S pit pattern
NBI	Nice type 1 No prominent vessels	Nice type 1 Dark spots Varicose microvascular vessels SSL-D: Nice type 2	Nice type 2 Leaf vein-like Expanded, brown capillary vessels

### 3.2 Classification of CRCs

Although the progression of serrated lesions into colorectal cancers (CRCs) is a common occurrence, it is important to note that CRCs originating from different serrated lesions exhibit distinct molecular characteristics and prognoses. Therefore, the classification of CRCs becomes imperative in facilitating clinical decision-making. At the molecular level, there exist two primary classification systems for CRCs: the Consensus Molecular Subtypes (CMS1 MSI Immune, CMS2 Canonical, CMS3 Metabolic, and CMS4 Mesenchymal) and the Colorectal Cancer Intrinsic Subtypes (CRIS-A, CRIS-B, CRIS-C, CRIS-D, CRIS-E) (52–54).

Among them, the CMS tumor classification, a widely accepted classification for CRC, has been demonstrated to effectively categorize CRC patients into distinct prognostic subgroups (55). In a study conducted, it was observed that CMS1 polyps exhibited a higher prevalence in the right colon among both sporadic and hereditary cohorts. Conversely, CMS2 polyps were found to be more prevalent in the left colon among both sporadic and hereditary cases. The findings of this study indicate that CMS1 carcinomas are likely to originate predominantly from HPs and SSLs, which are commonly observed in the right colon. It is noteworthy that the majority of HP and SSLs exhibit CMS1 characteristics, and they may undergo transitions into different CMS groups as they progress into carcinomas (56).

Indeed, the presence of SSL and HP is associated with an enrichment of both CMS1-like and CMS4-like phenotypes. Serrated lesions that advance through the MSI-high pathway are commonly categorized as CMS1. Conversely, serrated lesions that advance via the MSS pathway typically evolve into CMS4 cancers, characterized by an immunosuppressive microenvironment that promotes tumor invasion, immune evasion, and unfavorable survival outcomes (19, 56).

In 2015, Phipps et al. proposed a CRC classification of five molecular subtypes on the bases of (the tumor marker) as **Table 3** (57, 58).

**Table 3:** The classification of CRCs.

	Molecular characteristics	Precursor Lesions	CMS classification
Type1	BRAF-mutated, CIMP-H, MSI	SSLs with MLH1 methylation	CMS1
Type2	BRAF-mutated, CIMP-H, MSS	BRAF-mutated TSAs, SSLs without MLH1 methylation	CMS4
Type3	KRAS-mutated, CIMP-L, MSS	distally located KRAS-mutated TSAs, adenoma	CMS3
Type4	BRAF(-), KRAS(-), CIMP-L,MSS	develops through the adenoma–carcinoma pathway	CMS2(mainly),CMS3,CMS1
Type5	BRAF(-), KRAS(-),CIMP-H,MSI	affected with Lynch syndrome	CMS2(mainly),CMS4,CMS1,CMS3

In accordance with prognostic characteristics, the ranking of CRC subtypes is as follows: type 1, type 5, type 4, type 3, and type 2. The CIMP-H phenotype has demonstrated a significant correlation with female gender, proximal location, and advanced age. BRAF-mutated CRCs exhibiting CIMP-H and MSI are predominantly found in the right colon and exhibit distinctive histological features such as medullary, mucinous, and signet ring. These subtypes generally exhibit a favorable prognosis and display sensitivity to immune checkpoint blockage (ICB) therapy. In contrast, MSS CRCs are often characterized by poor differentiation and mucinous features, along with the presence of signet ring cells. These tumors consistently display resistance to treatment and a propensity for metastasis, resulting in a bleak prognosis. Consequently, when one SSL exhibits MLH1 methylation, it indicates a heightened likelihood of advancing into an advanced lesion, yet it is anticipated to have a more favorable prognosis (9, 12, 20, 33).

Furthermore, it is imperative to recognize that the CMS classifications do not directly correspond with the classifications based on tumor markers as mentioned above. Nevertheless, there exists compelling evidence indicating a certain level of similarity between CMS1 and Type 1 tumors, as well as between CMS3 and Type 3 tumors. Future research should investigate the collective impact of gene expression and tumor marker characteristics on the survival outcomes of patients diagnosed with colorectal cancer (58).

### 3.3 Endoscopic resection of SPs

Considering the potential malignancy of SSLs and TSAs, as well as the possibility of HPs progressing into these lesions, it is advisable to perform complete removal of all SPs during colonoscopy, with the exception of small HPs (<5cm) located in the sigmoid or rectum. Nevertheless, in cases of multiple diminutive rectosigmoid lesions, random biopsies should still be conducted to exclude the presence of more advanced SPs (34).

For small serrated lesions (<10 mm), hot snare polypectomy is found to be more effective and less prone to bleeding than cold forceps polypectomy. However, cold snare polypectomy (CSP) is highly recommended due to its lower risk of delayed post-polypectomy bleeding and perforation, shorter procedure times, and lower costs. Moreover, piecemeal CSP has been proven to be a safe and effective method for removing large serrated lesions (≥ 10 mm). The residual rates of ≥ 10 mm serrated lesions resected by CSP were lower compared to those of adenomas (9).

Endoscopic mucosal resection (EMR) or endoscopic submucosal resection (ESD) offer enhanced resection depth, improved pathological evaluation, and reduced recurrence rates in cases of SSL-Ds or suspicious lesions, as compared to CSP. EMR poses technical challenges when dealing with lesions larger than 20 mm. Conversely, ESD enables a thorough pathological evaluation and exhibits a superior initial cure rate, albeit necessitating advanced endoscopic expertise and incurring higher expenses (34, 59).

Endoscopic piecemeal mucosal resection (EPMR) is considered a secure technique for the removal of large polyps (≥ 20 mm) in medical practice. Nevertheless, its utilization is a subject of controversy due to the notable recurrence rate associated with this procedure (59).

In the management of SPS, it is crucial to prioritize the complete removal of all clinically relevant polyps, followed by rigorous endoscopic surveillance. This is due to the heightened risk (16-29%) of CRCs observed in patients with SPS. It is recommended to remove all polyps measuring ≥ 5 mm, as well as any polyps of any size that exhibit optical suspicion of dysplasia (47).

### 3.4 Surveillance recommendation

Prior to offering surveillance recommendations, it is imperative to conduct an assessment of the patient’s risk factors. As previously stated, the occurrence of SPs is correlated with various factors such as age, race, and alcohol consumption. When considering the combination of risk factors and polyp characteristics, particular attention should be given to specific demographic groups, including individuals aged 50 years or older, those with polyps located in the cecum, ascending colon, or transverse colon, and those with larger polyp sizes exceeding 5 mm. A study has indicated that individuals within this group often exhibit CIMP-H and MLH1 methylation in SSLs, indicating a heightened likelihood of progression into CRCs (40).

Interval CRC is a type of CRC that remains undetected during colorectal screening but is subsequently identified before the next scheduled screening date. The prevalence of interval CRCs occurring three years after colonoscopy ranges from 3.4% to 9.0%, with the majority of cases attributed to SSLs (22, 60, 61). In order to minimize the incidence of interval colorectal cancers (CRCs) and enhance the rate of polyp detection, it is imperative to prioritize appropriate bowel preparation, extended withdrawal times (preferably exceeding 6 minutes), and the expertise of the colonoscopist. These factors play a crucial role in ensuring timely and precise identification.16 Furthermore, the implementation of chromoendoscopy, NBI, and other techniques such as the utilization of mucosal exposure devices or antispasmodic agents can contribute to the identification and removal of lesions. Nevertheless, it is crucial to consider the potential implications of increased time and financial resources associated with these approaches.29 The optimization of polyp detection and differentiation can also be facilitated by the development of artificial intelligence systems. Current evidence indicates that a population undergoing screening colonoscopy should anticipate a detection rate of SP ≥15% (13, 62).

The duration of surveillance following polypectomy is determined by the size, number, and type of SPs as indicated in **Table 4**. Routine screening is recommended for most proximal or distal HPs measuring less than 10mm. For SSLs measuring less than 10mm, the surveillance period is 5-10 years for 1-2 SSLs, 3-5 years for 3-4 SSLs, and 3 years for 4-10 SSLs. The subgroup of patients who should undergo colonoscopy every 3 years includes those with any HPs or SSLs larger than 10mm, any SSL-Ds, any TSAs, or those with high-risk factors (63, 64).

**Table 4:** Surveillance time.

		Surveillance Time
HPs	HP<10mm	Routine Screening
	HP>10mm	3 years
SSLs	1-2 SSLs<10mm	5-10years
	3-4 SSLs<10mm	3-5 years
	4-10 SSLs<10mm	3 years
	SSLs >10mm	3 years
SSL-Ds		3 years
TSAs		3 years
after EPMR of SP >20mm		6 months
SPS	≥1 advanced polyp or ≥5 non-advanced polyps	1 year
	any other situation	2 years

For SPS, once cleared of all relevant lesions, surveillance advice is based on findings during the last colonoscopy. A surveillance interval of 1 year is advised after the resection of ≥1 advanced polyp or ≥5 non-advanced clinically relevant polyps. A surveillance advice of 2 years is advised in any other situation. The stopping rule for end of surveillance was not defined (47).

In the case of SPS, after the removal of all pertinent lesions, recommendations for surveillance are determined by the observations made during the most recent colonoscopy. Following the resection of one or more advanced polyps or five or more non-advanced clinically significant polyps, a surveillance interval of one year is recommended. In all other circumstances, a surveillance interval of two years is advised. The criteria for discontinuing surveillance were not specified (31).

It is noteworthy that a significant proportion, approximately 50%, of patients diagnosed with SSLs also exhibit adenomas during the same examination. Individuals presenting synchronous SPs alongside high-risk adenomas are at an elevated risk of developing metachronous CRCs. Consequently, it is advisable to reduce the surveillance interval for this particular subgroup of patients (65).

## 4 Conclusion

This article provides a comprehensive overview of the histologic and molecular characteristics, as well as the detection and management strategies, pertaining to serrated polyps. It is worth noting that serrated polyps exhibit a more rapid progression to cancer compared to traditional adenomas, thus contributing significantly to the incidence of early-onset colorectal cancers. While there exist numerous comprehensive surveillance guidelines for intestinal polyps, the absence of a specific unified guideline for serrated polyps necessitates further research in this area. Consequently, there is a need for more extensive investigations on serrated polyps, as well as the development of comprehensive and detailed surveillance guidelines.

## Author contributions

JW: Writing – original draft, Writing – review & editing. GX: Writing – original draft, Writing – review & editing. XH: Writing – review & editing. WL: Writing – review & editing. NY: Writing – review & editing. FH: Writing – review & editing. YZ: Funding acquisition, Writing – review & editing. JQ: Funding acquisition, Writing – review & editing.
